# An Integrated Pharmacological Counselling Approach to Guide Decision-Making in the Treatment with CDK4/6 Inhibitors for Metastatic Breast Cancer

**DOI:** 10.3389/fphar.2022.897951

**Published:** 2022-07-22

**Authors:** Rossana Roncato, Lorenzo Gerratana, Lorenza Palmero, Sara Gagno, Ariana Soledad Poetto, Elena Peruzzi, Martina Zanchetta, Bianca Posocco, Elena De Mattia, Giovanni Canil, Martina Alberti, Marco Orleni, Giuseppe Toffoli, Fabio Puglisi, Erika Cecchin

**Affiliations:** ^1^ Experimental and Clinical Pharmacology Unit-CRO Aviano, National Cancer Institute, IRCCS, Aviano, Italy; ^2^ Department of Medical Oncology-CRO Aviano, National Cancer Institute, IRCCS, Aviano, Italy; ^3^ Department of Medicine (DAME), University of Udine, Udine, Italy

**Keywords:** personalized medicine, TDM, polymorphisms, pharmacological counselling, breast cancer, CDK4/6 inhibitors

## Abstract

A wide inter-individual variability in the therapeutic response to cyclin-dependent kinases 4 and 6 inhibitors (CDKis) has been reported. We herein present a case series of five patients treated with either palbociclib or ribociclib referred to our clinical pharmacological counselling, including therapeutic drug monitoring (TDM), pharmacogenetics, and drug–drug interaction analysis to support clinicians in the management of CDKis treatment for metastatic breast cancer. Patients’ plasma samples for TDM analysis were collected at steady state and analyzed by an LC-MS/MS method for minimum plasma concentration (C_min_) evaluation. Under and overexposure to the drug were defined based on the mean C_min_ values observed in population pharmacokinetic studies. Polymorphisms in selected genes encoding for proteins involved in drug absorption, distribution, metabolism, and elimination were analyzed (*CYP3A4*, *CYP3A5*, *ABCB1, SLCO1B1*, and *ABCG2*). Three of the five reported cases presented a CDKi plasma level above the population mean value and were referred for toxicity. One of them presented a low function *ABCB1* haplotype (*ABCB1-rs1128503*, *rs1045642*, and *rs2032582*), possibly causative of both increased drug oral absorption and plasmatic concentration. Two patients showed underexposure to CDKis, and one of them was referred for early progression. In one patient, a CYP3A5*1/*3 genotype was found to be potentially responsible for more efficient drug metabolism and lower drug plasma concentration. This intensified pharmacological approach in clinical practice has been shown to be potentially effective in supporting prescribing oncologists with dose and drug selection and could be ultimately useful for increasing both the safety and efficacy profiles of CDKi treatment.

## 1 Introduction

Cyclin-dependent kinases 4 and 6 inhibitors (CDKis) in association with endocrine therapy represent the first- or second-line treatment of choice for hormone receptors (HR)-positive, HER2-negative metastatic breast cancer (MBC) patients (Giuliano et al., 2019; Schettini et al., 2020). Despite evidence of efficacy in terms of progression-free survival (PFS) and overall survival (OS), wide inter-individual variability regarding the therapeutic benefit of CDKis has been reported (Groenland et al., 2020), with some individuals experiencing increased and unexpected toxicity leading to dose adjustments, treatment delays, or discontinuations and other differential benefits.

**FIGURE 1:**
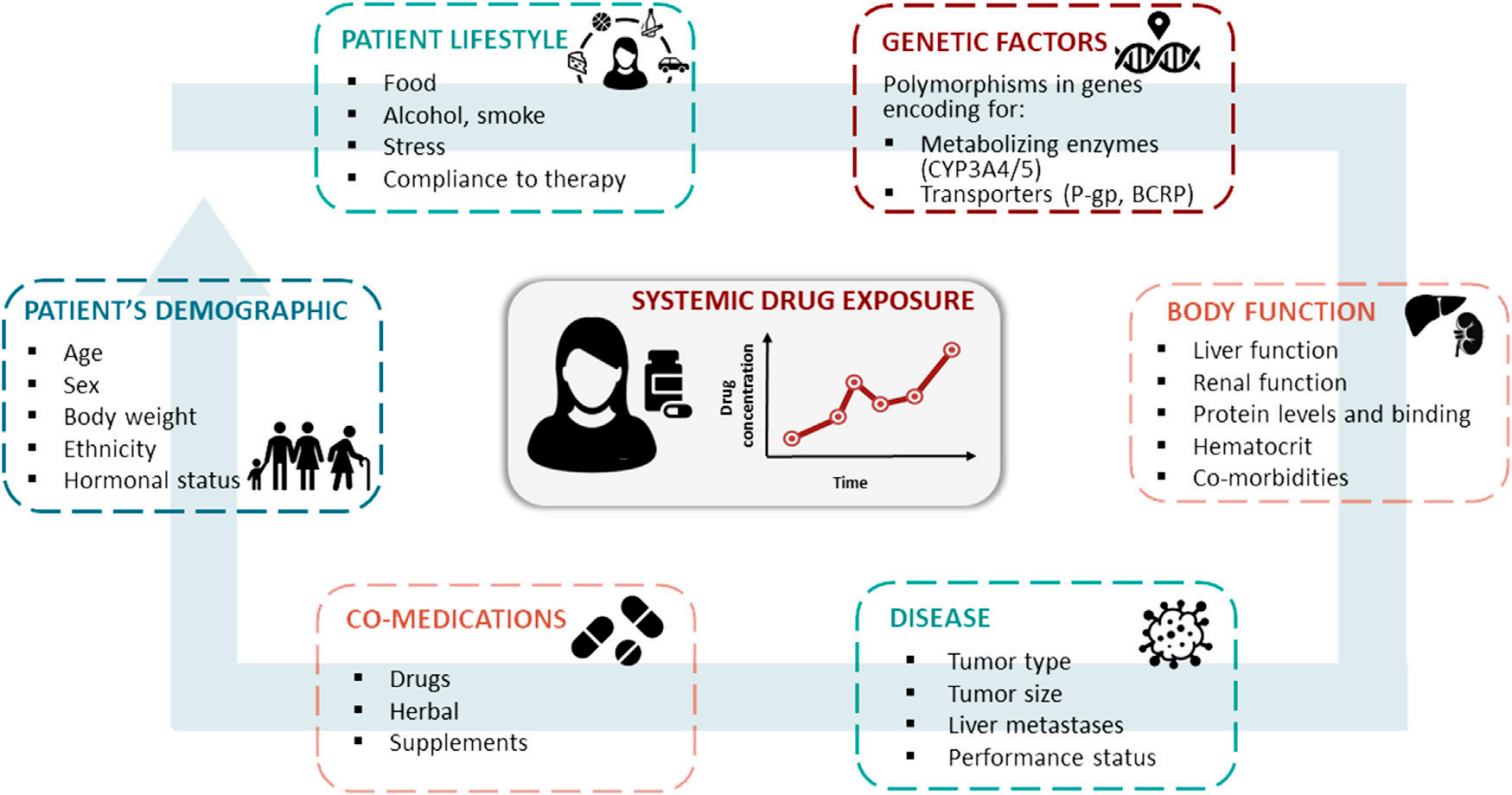
Patient’s characteristics, co-medications, lifestyle, genetic profile, body function, and disease are the main sources for inter- and intra-patient variability in the pharmacokinetics of oral targeted therapies, such as CDK4/6 inhibitors.

Among the factors that could be responsible for this phenomenon, differences in patients’ plasmatic exposure to the drugs should be considered. Indeed, the three CDKis approved for clinical use (i.e. abemaciclib, palbociclib, and ribociclib) exhibit considerable inter-individual variability in plasma exposure, with coefficients of variation of the minimum plasma concentration (C_min_) ranging from 40 to 95% (Groenland et al., 2020). The association between plasmatic exposure to CDKis and response to treatment is still poorly documented, but there is growing evidence of the relationship between exposure and toxicity (Groenland et al., 2020). A significant increase in the risk of neutropenia and thrombocytopenia has been reported in patients with higher palbociclib exposure in relation to the area under the plasma concentration-time curve (Schwartz et al., 2011; Flaherty et al., 2012; Verheijen et al., 2017). With respect to ribociclib, cardiac toxicity was associated with maximum plasma concentration (C_max_) at steady state (FDA, 2021b). An association between a higher C_min_ of ribociclib and the occurrence of hematological adverse events, such as neutropenia and thrombocytopenia, was also reported in a phase I study (Infante et al., 2016), with this trend confirmed in later studies (Groenland et al., 2020). A higher abemaciclib exposure was associated with a higher risk of neutropenia (US Food and Drug Administration, 2017a). However, concerning the exposure-efficacy relationship, more controversial results have been reported for both palbociclib and ribociclib (FDA, 2021b). In the PALOMA-1 clinical trial, a trend toward prolonged PFS was reported in patients with an average palbociclib concentration above the median population value (61 ng/ml) (Center for Drug Evaluation and Research, 2015). Contrarily, higher abemaciclib concentrations were associated with higher tumor shrinkage rate and lower hazard for disease progression in a dynamic PFS model on MONARCH 3 population (US Food and Drug Administration, 2017a). Several endogenous and exogenous factors may influence individual exposure to CDKis, including the patient’s genetic makeup and concurrent interacting pharmacological agents, which may affect drug absorption, distribution, metabolism, and excretion (ADME) efficiency (Figure 1).

After rapid absorption and distribution, all three CDKis undergo CYP3A-mediated metabolism. In addition to CYP3A, palbociclib is also metabolized in the liver by the sulfotransferase enzyme SULT2A1. Palbociclib and ribociclib undergo a glucuronidation reaction by the second-phase enzyme UGT. Palbociclib and abemaciclib are substrates of the efflux transporters P-glycoprotein (P-gp) and breast cancer resistance protein (BCRP), which may affect the bioavailability and distribution of the drug. Ribociclib is also a substrate of P-gp but shows no noticeable transport by BCRP.

The presence of polymorphisms affecting the expression level or functionality of enzymes involved in liver oxidative metabolism and intracellular transport could be considered predictive markers of CDKis exposure (Roncato et al., 2020).

CDKis have been introduced into clinical practice relatively recently, and there are currently little data on the potential use of pharmacogenetics to optimize their prescription. However, we have learned from other better studied gene-drug interactions such as *DPYD*-fluoropyrimidines, *CYP2D6*-tamoxifen, *TPMT/NUDT15*-thiopurines, and *UGT1A1*-irinotecan (Roncato et al., 2021), that genetic variability in ADME-related genes may be predictive of plasmatic drug exposure and clinical outcome. Similar exploratory results are available for some oral kinase inhibitors (KIs) as imatinib (Gardner et al., 2006), gefitinib (Li et al., 2007), sunitinib (Diekstra et al., 2014), and the selective estrogen modulator tamoxifen (Baxter et al., 2014) Therefore, it is tempting to hypothesize that similar effects could be observed for CDKis, but dedicated studies are needed. Drug–drug interactions (DDIs) were already demonstrated to significantly alter the pharmacokinetic profile of CDKis, thus influencing their safety and efficacy profile (Hoffman et al., 2016; Samant et al., 2020). AMBORA trial proved useful in the impact of pharmacological care on medication safety and patient-reported outcomes also from palbociclib and ribociclib treatment (Dürr et al., 2021).

Recently, the Experimental and Clinical Pharmacology Unit of the National Cancer Institute CRO Aviano implemented a pharmacological counselling service. The proposed counselling integrates therapeutic drug monitoring (TDM), patient pharmacogenetic profile, and co-medications management for a variety of drugs. These include drugs with an established exposure-response relationship and a validated target plasma C_min_, as in the cases of imatinib and sunitinib, for which TDM is recommended, or letrozole for which TDM is considered potentially useful (Mueller-Schoell et al., 2021). Regardless, the counselling is also provided for other TKIs for which there is inconclusive evidence of an exposure-response relationship and for which TDM is considered exploratory, as in the case of CDK4/6 and PARP inhibitors.

In the clinical setting of breast cancer treatment, it seems clear how beneficial an intensified pharmacological approach could be considering the impact of TDM, pharmacogenetics, and DDIs on CDKIs treatment outcome. Hematological adverse events are indeed the main dose-limiting toxicities associated with CDK4/6 inhibition and the reason for treatment suspension in about 70% of patients and for early dose reduction in 40–50% of patients (Braal et al., 2021). Abemaciclib exerts less hematologic toxicity compared with palbociclib and ribociclib. The most common reason for dose adjustment for abemaciclib is actually diarrhea. Such perspective could be helpful either in a reactive setting investigating under and overexposure to CDKis as potential surrogates for explaining unexpected treatment outcomes in terms of either toxicity or inefficacy, or in a pretherapeutic setting to personalize treatment and minimize exposure to DDIs and drug-gene interactions (DGIs). The present report describes the results of pharmacological counselling recently implemented at the National Cancer Institute, CRO Aviano, Italy, to support decision-making in MBC treatment with CDKis.

## 2 Patients and Methods

### 2.1 Patients

In September 2020, pharmacological counselling provided by the Clinical Pharmacology Unit of the National Cancer Institute CRO Aviano was opened to support medical oncologists in decisions making for first- or second-line CDKis (palbociclib, abemaciclib, and ribociclib) and endocrine therapy (letrozole) for HR-positive/HER2-negative MBC patients. Our counselling service was already available for other KI used in oncology as imatinib, sorafenib, regorafenib, sunitinib, lenvatinib, and three PARP inhibitors: olaparib, niraparib, and rucaparib. Written informed consent was obtained for pharmacogenetic and TDM analyses and the publication of the here presented reports. Any potentially identifying information was omitted. Data concerning age, disease, stage, and molecular profiling, treatment regimen and setting, drug dose, adverse drug reactions, and coadministered treatments were retrieved from the electronic medical record upon patients’ reporting by the prescribing medical oncologist. Toxicities were retrospectively collected through clinical records revision and graded according to Common Terminology Criteria for Adverse Events (CTCAE) Version 5.0.

The pharmacology laboratory is undergoing the UNI EN ISO-15189 accreditation program and is certified according to EMQN (www.emqn.org) and SKML (www.skml.nl) proficiency testing schemes for pharmacogenetic and TDM routine diagnostics, respectively.

### 2.2 Pharmacogenetic Analysis

Candidate genes were selected based on a literature search (PubMed-MEDLINE) focusing on those encoding for proteins involved in CDKis ADME (Roncato et al., 2020). Considering that CDKis are often administered in association with letrozole, SLCO1B1*5/*15/*17 was also genotyped (Gregory et al., 2017). Patients were genotyped for *CYP3A4* (*1B, rs2740574; *1G rs2242480; *3, rs4986910; *20, rs67666821; *22, rs35599367; *26, rs1381053638); *CYP3A5* (*3, rs776746; *6, rs10264272; *7, rs41303343); SLCO1B1 *5/*15/*17 (rs4149056); *ABCB1* (1236C > T, rs1128503; 3435C > T, rs1045642; 2677G > T/A, rs2032582); and *ABCG2* (421C > A, rs2231142). The pharmacogenetic analysis was performed by SNPline PCR Genotyping System platform employing Kompetitive allele-specific PCR (KASP) assays (LGC Genomics, Hoddesdon, United Kingdom) according to manufacturer’s instructions (Biosearch Technologies, 2021). Regarding *ABCB1* 2677G > T/A the tri-allelic discrimination was assessed using Pyrosequencing technology by PyroMark Q48 (Qiagen, Hilden, Germany). Primer sequences and genotyping details are available upon request. Positive and negative control samples were included in each analysis.

### 2.3 Therapeutic Drug Monitoring

Plasma was obtained by centrifugation at 2,450 g for 10 min at 4°C of whole blood EDTA tubes and stored at −80°C until analysis. Patients’ samples were analyzed with a newly developed LC-MS/MS method as previously reported (Posocco et al., 2020). Drug concentration was usually evaluated at specific time points, which allowed the evaluation of C_min_ or C_max_ at steady state. Patients were asked to have their last drug intake 24 h (C_min_), or 1–4 h (C_max_ of ribociclib) before the sampling time. Last administration (self-reported) and sampling times were also recorded. According to the literature (Verheijen et al., 2017), the average exposure of the approved efficacious dose was used as a proxy of target C_min_ and will be referred to as “target C_min_” in this article. In more detail, patients’ concentrations were compared with the reported population mean C_min_ of 61 ng/ml (US Food and Drug Administration, 2017b) for palbociclib at the standard dose of 125 mg/day and with the reported mean C_min_ of 732 ng/ml (FDA, 2021b) for ribociclib at the standard dose of 600 mg/day. The mean steady-state population C_max_ for ribociclib is 2,237 ng/ml. For letrozole, a C_min_ target value has already been proposed at 85.6 ng/ml by dedicated exposure-efficacy studies (FDA, 2021a).

### 2.4 Drug–Drug Interaction Analysis

Potential DDIs were identified using Lexicomp (UpToDate, 2021), Drug Interactions Checker on Drugs.com (Drugs.com, 2021), Flockhart Interaction Table (Flockhart et al., 2021), a summary of each coadministered product characteristics (EMA) (EMA, 2021b, EMA, 2021a) and Medscape (Medscape, 2021). All potential DDIs were analyzed and classified based on their clinical impact as moderate (pharmacological effects must be controlled) or severe (drug combination should be avoided).

### 2.5 Statistical Analysis Section

Descriptive statistics were used to present and analyze the TDM data. The mean population C_min_ value reported in the literature for patients treated with the standard dose was used to evaluate the TDM target, and the ±20% intervals were calculated. This interval was based on acceptable variability of analytical data according to incurred sample reanalysis criteria (FDA, 2018; EMA, 2022). Patients who fell outside this range were considered to be potentially under or overexposed to the drugs. For palbociclib, concentrations within 49–73 ng/ml and for ribociclib C_min_ within 586–878 ng/ml were considered to be within the range, regardless of the dose patients received.

## 3 Case Series

**FIGURE 2:**
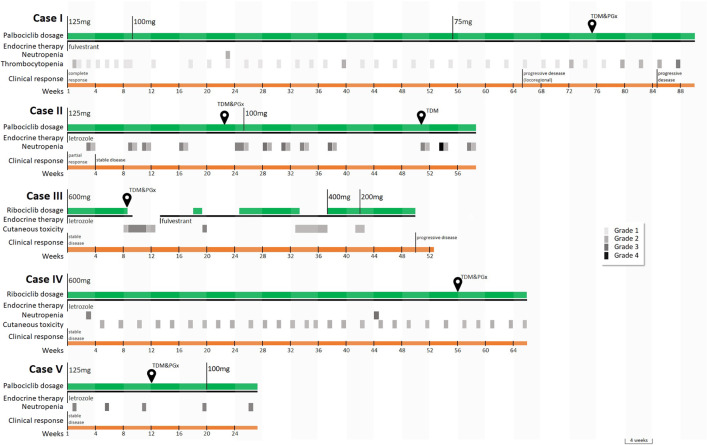
Timeline of the main events and sample collection for case series’ patients. Temporary postponements of therapy scheduled by the drug data sheet upon the occurrence of toxicity are not displayed. Legend: CR, complete response; PD, progressive disease; PR, partial response; SD, stable disease; TDM, therapeutic drug monitoring; PGx, pharmacogenetic analysis. Toxicities were graded according to Common Terminology Criteria for Adverse Events (CTCAE) Version 5.0.

At the time of publishing, more than 80 patients underwent the intensified pharmacological program. The five patients described were selected since they were of specific pharmacological interest and highlighted the opportunity offered by pharmacological counselling in the clinical interpretation of the cases. Patients’ characteristics, best response, treatment duration, major adverse drug reactions (ADRs), and metastatic sites at treatment start are summarized in Table 1 and events are visually represented in Figure 2.

**TABLE 1:** Demographic and clinical characteristics of included patients. Toxicities were reported according to NCI-CTCAE v5.0.

Parameter	Case I	Case II	Case III	Case IV	Case V
Age	72	71	75	52	60
Body mass index (kg/m^2^)	27.6	30.1	30	22.8	24.9
ANC baseline	3.03 × 10^3^/mm^3^	3.14 × 10^3^/mm^3^	N.A.	7.21 × 10^3^/mm^3^	2.39 × 10^3^/mm^3^
Index drug and dose	Palbociclib capsules 75 mg/day + fulvestrant 500 mg Q28	Palbociclib capsules 125 mg/day–>100 mg/day + letrozole 2.5 mg	Ribociclib 200 mg/day + Letrozole - > fulvestrant 500 mg Q28	Ribociclib 600 mg/day + letrozole 2.5 mg	Palbociclib capsules 125 mg/day–>100 mg/day + letrozole 2.5 mg
Co-administered drugs	Codeine/paracetamol as needed; duloxetine 30 mg/day; venlafaxine 150 mg/day; and vitamin D supplement	Aspirin 100 mg/day; phlehydrin 200 mg/day; pantoprazole 20 mg/day; allopurinol 150 mg/day; ezetimibe/simvastatin 10 mg/10mg day; hydrochlorthiazide 12 mg/day; and vitamin D supplement	Aspirin 100 mg day; atenolol 100 mg/day; atorvastatine 40 mg/day; levothyroxine 75 mg/day; and omeprazole 20 mg/day	Pantoprazole 20 mg/day	Zolendronic acid 4 mg/ev every 12 weeks
Setting	II line	I line	I line	I line	I line
Metastatic sites	Bones and lymph nodes	Lung, bones, and lymph nodes	Lung	Lung, bones, and lymph nodes	Liver, bones, and lymph nodes
Clinical inquiry	Evaluation of DDIs with antidepressant therapy in suspected disease progression	Recurrent grade 3 neutropenia	Persistent cutaneous toxicity for more than 1 year	Recurrent toxicity	Neutropenia
Maximum toxicity grade	Neutropenia G3; piastrinopenia G3	Neutropenia G4	Cutaneous rash G3	Neutropenia G3; cutaneous rash G1	Neutropenia G3
*CYP3A4* phenotype	NM (*1/*1)	NM (*1/*1)	NM (*1/*1)	NM (*1/*1)	NM (*1/*1G)
*CYP3A5* phenotype	PM (*3/*3)	PM (*3/*3)	PM (*3/*3)	PM (*3/*3)	IM (*1/3)
*ABCB1* profile	*1236CC*; *3435CC*; *2677GG*	*1236TT*; *3435TT*; *2677TT*	*1236CT*; *3435CT*; *2677GT*	*1236CT*; *3435CT*; *2677GT*	*1236CT*; *3435CT*; *2677GT*
*ABCG2* profile	*421CC*	*421CA*	*421CC*	*421CC*	*421CC*
*SLCO1B1* profile	*1/*5 decreased function	*5/*5 poor function	*1/*1 normal function	*1/*5 decreased function	*1/*1 normal function
TDM values, C_min_ values (drug and dosage)	27.3 ng/ml (palbociclib 75 mg/day)	85.2 ng/ml (palbociclib 125 mg/day), 62.3 ng/ml (palbociclib 100 mg/day), and 93.1 ng/ml (letrozole 2.5 mg/day)	1,100 ng/ml (ribociclib 600 mg/day) and 70.3 ng/ml (letrozole 2.5 mg/day)	1717.6 ng/ml (ribociclib 600 mg/day) and 181.9 ng/ml (letrozole 2.5 mg/day)	36.2 ng/ml (palbociclib 125 mg/day) and 31.4 ng/ml (letrozole 2.5 mg/day)
Pharmacological counselling indication	Continue with current antidepressant treatment. Consider switching to abemaciclib	Severe neutropenia could be due to overexposure and other risk factors. Dose reduction is safe	Cutaneous toxicity could be due to drug overexposure. Dose reduction is safe	Hematologic and recurrent cutaneous toxicity could be due to overexposure. Dose reduction is safe	Neutropenia in an underexposed patient with risk factors for neutropenia development. Dose reduction may not be decisive; consider switching to abemaciclib
Patient’s clinical outcome	Palbociclib treatment failure due to disease progression	The palbociclib dose was reduced, but neutropenia persisted	The dose of ribociclib was reduced after more than 1 year of intermittent treatment due to toxicity. Ribociclib treatment ultimately failed	The patient continued to develop hematologic and cutaneous toxicities at standard dosage. Ultimately toxicity was considered tolerable, and the dose was not reduced	The palbociclib dose was reduced with no improvement in toxicity

ANC, absolute neutrophil count.

### 3.1 Case I

At the age of 54, Case I was diagnosed with breast cancer and, after neoadjuvant chemotherapy, underwent a radical mastectomy followed by adjuvant endocrine therapy with tamoxifen for 5 years and with letrozole for another year. Five years later, after a diagnosis of nodal recurrence, letrozole was reintroduced as first-line therapy. After 2 years, because of bone and nodal progression, second-line therapy with fulvestrant and palbociclib was started. In the most recent period, she started venlafaxine treatment due to a moderate depression status in addition to duloxetine, already prescribed to treat a mood disorder, and a mild bone progressive disease (PD) was reported at the subsequent positron emission tomography (PET) scan. The woman was referred to pharmacological counselling to better characterize the potential impact of DDIs between palbociclib and venlafaxine on the outcome of a CDKi treatment. By the time pharmacological counselling was required, palbociclib dosing had already been reduced (75 mg/day instead of 125 mg/day, 3 weeks on/1 week off), due to previous recurrent hematological toxicity (neutropenia and thrombocytopenia).

A blood sample was collected at steady state to assess the concentration of palbociclib in plasma 24 h after the last intake (C_min_). As specified earlier, the patient was receiving a daily dose of 75 mg at the time of blood sampling. The reported average C_min_ in patients treated at the standard dose of 125 mg/day palbociclib is 61 ng/ml. The measured C_min_, which was equal to 27.3 ng/ml was consistent with the reduced dose administered (75 mg/day). When analyzing the case from a pharmacogenetic point of view, no defective genetic variants affecting the metabolism or transport of palbociclib (in the genes *CYP3A4*, *CYP3A5*, *ABCB1*, and *ABCG2*) were highlighted.

The analysis of potential DDIs highlighted only an increased risk of serotonin syndrome/serotonin toxicity development due to coadministration of serotonin/norepinephrine reuptake inhibitors. Although this information is not strictly useful for the purposes of the requested pharmacological counselling, a potentially damaging DDI was highlighted further supporting the utility of intensified pharmacological care in this setting.

Possibly a prolonged underexposure to the active drug, among other pathological factors, could have affected treatment efficacy. It is also likely that the toxicity experienced by the patient was not related to overexposure to the drug but more likely to high sensitivity to the drug’s toxic effect related to other causes. The patient presented a baseline absolute neutrophil count (ANC) of 3.03 × 10^3^/mm^3^ that, according to previous reports, could predispose to a higher risk of neutropenia (Iwata et al., 2021). Based on our analysis, a switch to abemaciclib could have been considered due to a lower incidence of treatment-associated hematologic toxicity neutropenia (Sledge et al., 2020).

The bone lesion for which the patient was referred was treated with locoregional radiotherapy, and the patient continued treatment with fulvestrant and palbociclib. Subsequently, after 5 months, another mild bone PD was noted on the PET scan and locoregional radiotherapy was again performed and treatment continued. Finally, a systemic PD was registered 1 month later and second-line therapy with capecitabine was started. No more blood draws were collected for C_min_ quantification of palbociclib and letrozole.

**Key takeaway**: The pharmacological evaluation excluded an interaction between treatment with venlafaxine or duloxetine and palbociclib outcome, avoiding the necessity to modify the anti-depressive treatment. The counselling also ruled out the presence of pharmacogenetic variants and overexposure to palbociclib as a reason for the observed hematologic toxicity and highlighted the presence of a baseline ANC median value below 3.60 (× 10^3^/mm^3^) as a risk factor for it. The dose reduction put the patient at risk for sub-optimal exposure to the drug. Switching to a compound less associated with bone marrow suppression, such as abemaciclib, could have been a valuable strategy to overcome recurrent hematologic toxicity. Unfortunately, at the time of writing switch between CDKis was considered an off-label intervention.

### 3.2 Case II

Case II was diagnosed with breast cancer when she was 60 and at that time underwent conservative breast surgery, followed by 5-year adjuvant endocrine therapy with letrozole. Recently, a computed tomography (CT) scan has highlighted distant metastasis with lungs, lymph nodes, and bone involvement. First-line endocrine therapy was therefore started with palbociclib 125 mg/day (3 weeks on/1 week off) and letrozole 2.5 mg/day with the occurrence of recurrent neutropenia grade 3 for which the dose was reduced to 100 mg/day and the patient referred to the pharmacological counselling. Plasma concentration at steady state of both palbociclib and letrozole was determined at 22 h after the last drug assumption. The analysis showed: 1) a plasma concentration of 85.2 ng/ml of palbociclib treated at 125 mg/day (approximately 30% higher than the mean C_min_ of 61 ng/ml reported in the literature for treatment at 125 mg/day); 2) a plasma concentration of 93.1 ng/ml for letrozole, in line with the desired threshold (85.6 ng/ml). The pharmacogenetic analysis highlighted an *ABCB1* haplotype with the homozygous presence of *ABCB1* rs1128503; *ABCB1* rs1045642 and *ABCB1* rs2032582 resulting in P-gp protein low function/expression (Salama et al., 2006) that could be compatible with increased drug exposure, further corroborated by the heterozygous presence of ABCG2 421C > A (Morisaki et al., 2005). The patient also presents a SLCO1B1*5/*5 genotype associated with a poor function phenotype (Cpicpgx, 2015).

From our analysis no additional risk factors were present and our suggestion was to monitor treatment at a reduced dose. The advice was followed and palbociclib C_min_ was found to be 62.3 ng/ml, after a dose reduction to 100 mg/day, in line with the population target C_min_. Regardless, the patient developed again up to grade 4 neutropenia. The patient presented a baseline ANC of 3.14 × 10^3^/mm^3^.

**Key takeaway**: The pharmacological evaluation highlighted the presence of three risk factors for the development of neutropenia: 1) overexposure to 125 mg/day palbociclib; 2) baseline ANC median value below 3.60 (×10^3^/mm^3^); 3) low function *ABCB1* haplotype.

The pharmacological evaluation also highlighted the importance of TDM to exclude a causal link between overexosure and recurrent neutropenia, as this adverse reaction was still observed after reducing palbociclib dose. In fact, at 100 mg/day of palbociclib, systemic exposure was within the C_min_ target range with persisting toxicity.

### 3.3 Case III

Case III was diagnosed with breast cancer when she was 57 and underwent conservative breast surgery, followed by adjuvant chemotherapy and adjuvant endocrine therapy with tamoxifen. Sixteen years later, lung metastases were detected, and first-line endocrine therapy was started with ribociclib (600 mg/day, 3 weeks on/1 week off) and letrozole 2.5 mg/day. The treatment was well tolerated but after 2 months a persistent grade 2 skin rash was observed. After 1 year of intermitting treatment at full dosage with several treatment suspensions (up to 2 months), the patient was referred for pharmacological counselling.

Two samples were collected at steady state at 24 h, and an hour and a half from the last drug intake to evaluate ribociclib and letrozole plasma levels. The samples allowed an accurate assessment of the drug C_min_ and a hypothetical estimation of ribociclib C_max_ (reached between 1 and 4 h after drug assumption). C_min_ resulted to be 1,100 ng/ml for ribociclib and 70.3 ng/ml for letrozole, while the estimated C_max_ values were 2020 ng/ml and 94.1 ng/ml, respectively. Ribociclib concentration at 24 h after the last dose far exceeded the reported target population C_min_ of 732 ng/ml (US Food and Drug Administration, 2017a), suggesting a potential role in the development of skin toxicity, while letrozole C_min_ appeared slightly lower than the target population C_min_ of 85.6 ng/ml.

Neither the pharmacogenetic analysis, focusing on the search for defective polymorphisms in *CYP3A4, CYP3A5, SLCO1B1, ABCB1,* and *ABCG2*, nor the analysis of DDIs explained the patient’s overexposure to ribociclib. Because the drug’s package insert does not suggest a treatment strategy for the occurrence of skin toxicity, treatment with ribociclib was continued by switching from letrozole to fulvestrant and, a few months later, reducing the dose first to 400 mg and then to 200 mg. The patient developed less severe skin reactions afterward. However, after 4 months of treatment with ribociclib at a reduced dosage, a disease progression was reported to CT scan and second-line treatment with capecitabine was initiated.

Within the pharmacological counselling, TDM could have guided an earlier dose reduction to a more tolerated dosage, ensuring an adequate plasma exposure and avoiding an intermittent therapy that could have compromised treatment efficacy.

Key takeaway: The pharmacological evalutation highlighted an overexposure to ribociclib administered according to the standard regimen (600 mg/day) as a possible risk factor for the cutaneous toxicity and ruled out pharmacogenetic variants as a potential cause. TDM-guided early dose reduction to a more tolerated dose could have ensured adequate plasma exposure. Unfortunately, the counselling service was not made available until the patient had been treated for more than a year and recurrent episodes of toxicity had occurred.

### 3.4 Case IV

Case IV concerns a 38-year-old woman diagnosed with early breast cancer who, after neoadjuvant chemotherapy underwent radical mastectomy and adjuvant endocrine therapy with tamoxifen and a Luteinizing Hormone-Releasing Hormone analogue (LHRHa) for 5 years. Seven years later, after the diagnosis of tumor relapse with nodal metastases, first-line therapy with letrozole and ribociclib was started. The patient was referred to pharmacological counselling after several episodes of persistent skin dryness, rash, and swallowing difficulty, a low-grade allergic reaction probably associated with ribociclib. Two episodes of grade 3 neutropenia were also recorded throughout the treatment despite a baseline ANC of 7.21 × 10^3^/mm^3^. In the last 14 months, the patient was treated with ribociclib 600 mg/day (according to 3 weeks on/1 week off schedule) and letrozole 2.5 mg/day.

To evaluate ribociclib and letrozole plasma exposure at steady-state, a blood sample was collected approximately 23.5 hours after the last drug intake. Patient’s C_min_ resulted in being 1717.6 ng/ml for ribociclib and 181.9 ng/ml for letrozole. These concentrations largely exceeded the target population C_min_ values reported in the literature (i.e., 732 ng/ml for ribociclib and 85.6 ng/ml for letrozole).

Neither the pharmacogenetic analysis, with no defective polymorphisms in the *CYP3A4, CYP2C9, ABCB1* (T allele found in heterozygous form in the three analyzed loci), and *ABCG2* genes, nor the DDIs analysis explained the patient overexposure to the drug. The patient also presents a decreased function *SLCO1B1* genotype-predicted phenotype (SLCO1B1*1/*5).

A dose reduction could have been considered for that patient with the recommendation to monitor ribociclib plasma levels through TDM analysis to increase the chance of a safer treatment and to ameliorate treatment compliance.

Key takeaway: The pharmacological evaluation highlighted the presence of a risk factor for the development of neutropenia consisting of the overexposure to 600 mg/day ribociclib and ruled out the presence of pharmacogenetic variants as a cause. An early TDM-guided dose reduction could have been better tolerated by the patient and exposure to reduced dosages could have been monitored, but the patient was reactively referred to our service, after several episodes of toxicity.

### 3.5 Case V

Case V was diagnosed with luminal MBC with nodal and bone metastases, therefore a first-line therapy with palbociclib (125 mg/day, 3 weeks on/1 week off) and letrozole (2.5 mg/day) was started. After 3 months from initiation, pharmacological counselling was required to monitor therapy because of the underlying neutropenia. A blood sample was taken from the patient for the assessment of the concentration of palbociclib 23 h after the last drug intake (C_min_) at steady state. The reported average C_min_ in patients treated at the standard dose of palbociclib 125 mg/day is 61 ng/ml and 85.6 ng/ml for letrozole. The concentration of palbociclib found in Case V was 36.2 ng/ml, while letrozole C_min_ was 31.4 ng/ml therefore, both concentrations were lower than the target C_min_.

The pharmacogenetic analysis revealed no defective polymorphisms in the *CYP3A4, CYP2C9, SLCO1B1, ABCB1* (T allele found in heterozygous form in the three analyzed loci), and *ABCG2* genes except for a heterozygous CYP3A5*3/*1 genotype. The resulting *CYP3A5* intermediate metabolizer status could potentially be responsible for an accelerated metabolic inactivation of palbociclib which could, in turn result in reduced plasma concentration. The analysis of coadministered drugs revealed no potential DDIs. It was also verified that the drug was taken with food, excluding this additional source of variability for palbociclib capsules (Ruiz-Garcia et al., 2017).

After 5 months, grade 3 protracted neutropenia required a dose reduction of palbociclib from 125 mg/day to 100 mg/day despite the relatively low drug plasma level. Other factors not related to drug exposure could have been the cause of the neutropenia. It should be noted that the patient presented a baseline ANC of 2.39 × 10^3^/mm^3^, possibly concurring with toxicity development (Iwata et al., 2021). The patient developed again neutropenia grade 3. No more blood samples were collected for C_min_ quantification of palbociclib and letrozole. It was hypothesized that hypersensitivity of the patient to the toxic effect of the drug, unrelated to the pharmacokinetic profile of palbociclib, may have been the cause of the neutropenia. A switch to abemaciclib have been considered due to the lower incidence of treatment-associated hematologic toxicity, particularly neutropenia (Sledge et al., 2020).

**Key takeaway**: The pharmacological evaluation ruled out overexposure as a reason for the observed neutropenia and highlighted the presence of a baseline ANC median value below 3.60 (×10^3^/mm^3^) as a risk factor for it. The counselling also identified an underexposure to 125 mg/day palbociclib and the CYP3A5*1 allele as a potential risk factor for this. The dose reduction put the patient at risk for suboptimal exposure to the drug. A different strategy to manage recurrent hematologic toxicity, as switching to a compound less associated with bone marrow suppression, like abemaciclib, could have been considered.

## 4 Discussion

The present series reported five cases referred to CRO-Aviano pharmacological counselling during CDKis treatment to analyze potential innovative precision medicine strategies based on an integrated pharmacological approach to support clinical decision-making through CDKis TDM coupled with pharmacogenetic profiling and co-medication management.

Our intensified pharmacological counselling program initially included oral anticancer KIs, where TDM has a more consolidated impact as in the case of imatinib or sunitinib. Only recently CDKis have been included, as they represent a class of drugs that meets most of the characteristics necessary for drugs to be good candidates for TDM evaluations: narrow therapeutic window, high interpersonal variability in exposure, and evidence of exposure-response association. In addition, hematologic adverse events are dose-limiting toxicities for CDKis, that are often responsible for dose reductions and treatment delays. In clinical practice, dose adjustments of CDKis are based solely on individual tolerability.

The purpose of this case series is to provide additional information on the potential validity of systematically offering an intensified pharmacology program for CDKis.

The multidisciplinary team involved in this service consists of three clinical pharmacology specialists, a pharmacist and a chemist expert in LC-MS/MS, a biologist specialized in pharmacogenetic variants, and a technician for the pharmacogenetic analyses.

Cases are referred by the oncologist to the Experimental and Clinical Pharmacology Unit of CRO-Aviano in an unsystematic, and often reactive manner, to the onset of a clinical issue (i.e., the introduction of a new co-medication, unexpected toxicity, or progression). The laboratory receives the request, performs pharmacogenetic and TDM analysis, and returns the pharmacological counselling report within 1 month after the blood draw. Patients may be followed longitudinally, especially in case of changes in treatment (e.g., dosage, schedule, or addition of co-medication) or persistence of the clinical problem. An additional report is provided if needed or requested. The service is freely available to cancer patients upon medical prescription and is covered by the Italian healthcare system.

In our case series, two out of five patients (Cases I and V) were found to have CDKi plasma exposure (C_min_) below the population’s target value reported for the approved dose, while three out of five patients (Cases II, III, and IV) were above that. In this pilot setting, the TDM approach proved useful in distinguishing between patients who had toxicity on a pharmacokinetics basis (i.e., due to the overexposure to the drug) and could be successfully treated with lower doses and those who could not tolerate treatment despite exposure levels equal or below the target C_min_. When toxicity occurred in an underexposed or normally exposed patient, dose reduction based on toxicity was not only an ineffective measure to prevent the development of future toxicities but instead exposed the patient to suboptimal CDKis plasma concentrations.

TDM may be helpful in those patients treated at standard dose who show a poor response to the drug. In those cases, measuring the drug plasma concentration allows us to understand if lack of efficacy could be related to underexposure or not. If positive, compliance should be evaluated as well as genetic polymorphism or dangerous drug–drug interactions. In case I the exclusion of a dangerous DDI, allowed the physician to preserve the anti-depressant treatment that was suspected to be the cause of treatment inefficacy.

Since severe toxicities were observed in overexposed cases, an early TDM-guided dose reduction could have been beneficial in limiting toxicities while assuring adequate drug exposure without treatment interruptions. In this case series, TDM was proved helpful in a reactive setting to recurrent toxicities providing a rationale for dose reduction to treating physician when toxicity occurred in an overexposed patient.

The case series further supported the role of baseline ANC as the co-occurrence of high C_min_ levels and low ANC was observed in cases characterized by severe hematological toxicity (Iwata et al., 2021; Palmero et al., 2021). Patients with a high baseline ANC and a high C_min_ could be still at risk of neutropenia, as in Case IV, and benefit from a dose reduction coupled with TDM monitoring. On the other hand, patients with a low C_min_ and low baseline ANC, such as Case I and V, could benefit more from a drug switch rather than a dose reduction since the hematologic event was probably not related to overexposure to palbociclib. In such cases, an evidence-based rationale is provided for switching to abemaciclib, a CDKi characterized by a better hematologic safety profile.

Among the potential CDKis toxicities, cutaneous toxicity is an emerging topic that still needs to be fully elucidated in its etiology. Interestingly, cases III and IV developed cutaneous toxicity and were overexposed to ribociclib.

Multiple intrinsic and extrinsic factors can affect the plasma concentration of CDKis (Figure 1), such as a patient genetic profile for ADME genes and other concomitant pharmacological treatments which, could expose the patients to the risk of therapeutic inefficacy (due to underexposure) or increased toxicity (due to overexposure).

In this series, one out of three patients presenting plasma levels above the population target value (i.e., Case II), harbored functional variants in the gene coding for P-gp and BCRP mediated transport. Specifically, Case II was a carrier of the low function haplotype resulting from the combination of *ABCB1-rs1128503*, *rs1045642,* and *rs2032582* (Hoffmeyer et al., 2000; Horinouchi et al., 2002). A reduced P-gp expression in the gut is likely to increase oral absorption of the drug, potentially leading to an increased plasmatic concentration, as observed in this patient. Recently, the association between *ABCB1-rs1128503* genotype and palbociclib-related neutropenia risk was also confirmed by the pharmacogenetic analyses in PALOMA-2 and -3 (Iwata et al., 2021). Multivariate analysis showed a significant protective effect of *ABCB1-rs1128503* CC wild-type genotype in terms of neutropenia. However, the authors did not observe an association between any *ABCB1* genotype and palbociclib exposure. Some studies on other oral anti-cancer kinase inhibitors, such as imatinib, have demonstrated that the low functionality *ABCB1* haplotype, herein reported, was associated with a differential exposure to the drug, similar to what we observed (Harivenkatesh et al., 2017). The role of P-gp genetic variants on the pharmacokinetics of substrate drugs is still controversial (Wolking et al., 2015) and further studies are needed. An additional detrimental effect on drug absorption could be exerted by *ABCG2* 421C > A polymorphism, carried by the patient that has been already reported to negatively affect the transport efficiency of ABCG2 for several substrates *in vitro*, and the pharmacokinetics of gefitinib, *in vivo* (Li et al., 2007).

On the other hand, Case V was a carrier of the heterozygous CYP3A5*1/*3 genotype, compatible with an increased expression of CYP3A5 and with a more efficient palbociclib metabolism. This might explain the lower plasma concentration observed in case V. Similar findings were already reported for other oral anti-cancer drugs such as imatinib (Harivenkatesh et al., 2017).

Cases II, IV and V were treated also with letrozole. Interestingly, case V presented a low letrozole C_min_ concentration, suggesting that CYP3A5 intermediate metabolizer status could potentially be responsible also for letrozole accelerated metabolic inactivation. On the other hand, cases II and IV showed high C_min_ letrozole concentrations and carried at least one C-allele in SLCO1B1*5 (T521C) associated with low OATP1B1 activity. Accordingly, it has been previously reported that SLCO1B1*5 carriers have higher exposure to another aromatase inhibitor substrate, exemestane (Gregory et al., 2017).

DDIs significantly alter the pharmacokinetic profile of CDKis since the coadministration of pharmacological or dietary agents impacts the expression of relevant ADME genes such as *CYP3A4*, *CYP3A5,* or *ABCB1* and could strongly affect CDKis plasma concentration. This, in turn, could translate into an unexpected variability in drug response and toxicity (Bellet et al., 2019). None of the cases reported in this article presented significant pharmacological interactions allowing to explain either the reported clinical phenotype or the CDKis plasma levels detected. An integrated clinical pharmacology approach should always be considered in association with pharmacogenetic profiling to better define potential phenoconversion, and avoid conflicting results often observed in DGIs association studies (Shah and Smith, 2015; Hahn and Roll, 2021). Evaluation of DDIs that can compromise optimal drug exposure is part of clinical practice, although not systematically applied (Dürr et al., 2021; Leenhardt et al., 2021). Regardless, drug–drug–gene interactions resulting from the superimposition of a DDI on a DGI are often cause of phenoconversion of the genotype-predicted phenotype. Based on our experience as pharmacologists and physicians involved in this pilot counselling program, we would advise this service in the future to make informed CDKis dose-reduction or switch between them. Moreover, the effect of DDI could be better weighted and interfering co-medications avoided.

No reported patient was taking abemaciclib. Regardless, the proposed target C_min_ for abemaciclib is 181 ng/ml from MONARCH 3 patients referring to 132 mg/twice day (US Food and Drug Administration, 2017b).

Our study clearly has some limitations, mainly related to the small sample of treated patients, the retrospective collection of toxicity data, and the heterogeneity of the CDKis used. Because of these limitations, we were unable to examine meaningful associations between patients’ clinical and molecular characteristics and response to treatment. Moreover, it must be noted that the presented approach, focused on the pharmacokinetic/pharmacogenetic profiling of the patient with the aim to reach a target C_min_, is based on population pharmacokinetic results and does not consider the intrinsic pharmacodynamic/pharmacogenetic patient variability (Gerratana et al., 2021, 2021; Hertz et al., 2021). Additional research on this still uninvestigated source of variability should be warranted to highlight the pharmacological ground of cases of resistance or hypersensitivity to the drug, despite adequate plasma exposure, as those reported in this case series.

Although dedicated studies are needed to determine the clinical validity of this approach, based on the experience from this case series, we recommend this service as a valuable tool for evidence-based treatment with CDKis. Pharmacological counselling informed oncologists in the decision-making process, who could choose to manage specific toxicity by reducing the dose or switching to another CDKis. Moreover, the potential impact of DDI could be better weighted and futile changes to the co-administered drugs could be avoided in case of clinical irrelevance. Beyond dose individualization, integrated pharmacological counselling could also be useful for resource optimization, especially for expensive KIs such as CDKis. Value-based prescribing strategies for oral oncology drugs alone could save US $12 billion or more globally per year (Goldstein et al., 2020). Re-evaluating dosing strategies represents an opportunity to achieve significant value for patients both in terms of increasing safety and appropriate use of drugs and for healthcare systems in economic terms.

## Data Availability

The datasets for this article are not publicly available due to concerns regarding participant/patient anonymity. Requests to access the datasets should be directed to the corresponding authors.
